# Unveiling the Colors of Mustelids: A Historical Review on the Emergence of Chromatic Disorders and Their Ecological Effects in Mustelids Worldwide with Report of the First Case of Erythrism in Eira barbara (Carnivora, Mustelidae)

**DOI:** 10.3390/ani14233354

**Published:** 2024-11-21

**Authors:** Leonardo Cotts, Giovanna Costa, Sofia Isabel Gabriel, Oscar Rocha Barbosa, Caryne Braga

**Affiliations:** 1 Laboratório de Zoologia de Vertebrados–Tetrapoda (LAZOVERTE), Departamento de Zoologia, Instituto de Biologia Roberto Alcantara Gomes, Universidade do Estado do Rio de Janeiro (UERJ), Rua São Francisco Xavier, 524, PHLC 5º Andar, Sala 522A, Rio de Janeiro 27965-045, Brazil; obarbosa@uerj.br; 2 Centro de Ecologia, Evolução e Alterações Ambientais (cE3c), Faculdade de Ciências da Universidade de Lisboa, 1749-016 Lisboa, Portugal; 3 Instituto de Biodiversidade e Sustentabilidade (NUPEM), Universidade Federal do Rio de Janeiro (UFRJ), Avenida São José do Barreto, 764, São José do Barreto, Macaé, Rio de Janeiro 27965-045, Brazil; giosgcosta@gmail.com; 4 CESAM—Centro de Estudos do Ambiente e do Mar, Departamento de Biologia Animal, Faculdade de Ciências da Universidade de Lisboa, Campo Grande, 1749-016 Lisboa, Portugal; sigabriel@ciencias.ulisboa.pt; 5 Laboratório de Ciências Ambientais (LCA), Centro de Biociências e Biotecnologia (CBB), Universidade Estadual do Norte Fluminense Darcy Ribeiro (UENF), Campos dos Goytacazes, Rio de Janeiro 27965-045, Brazil

**Keywords:** animal pigmentation, anomalous coloration, camera trap survey, chromatic disorder, mammals

## Abstract

Mustelidae is the largest and most diverse family within the order Carnivora, encompasses 65 species and 22 genera, and is widely distributed around the world. Mustelids exhibit a multiplicity of coloration patterns, ranging from darker tones, such as black and brown, to lighter tones, such as pale yellow and white. Anomalous colorations in wild mustelids are occasionally reported, but these records are still poorly discussed in the scientific literature and are often subject to misidentification and/or incorrect terminology. Among mustelids, the Tayra, *Eira barbara*, is suggested to have the highest frequency of chromatic disorders reported in the formal scientific literature, although most cases involve hypopigmentation. Here, we report and describe the first case of a reddish-colored tayra, a rare chromatic disorder known as erythrism, and compare their coloration to that observed in the subspecies currently suggested for *E. barbara*. We also conducted a survey and a review of chromatic variants in mustelids based on the formal scientific literature, reporting 119 anomalously colored individuals from 1890 to 2024, and proposed accurate terminology for misidentified cases. Additionally, we discuss the occurrence of these chromatic disorders on a global scale and their potential ecological effect on wild mustelids.

## Simple Summary

Coloration in mammals has several functions, including concealment, communication with other individuals of the same species, social signaling to predators and prey, regulation of body temperature, and attraction for reproduction. However, environmental imbalances and/or occasional genetic mutations can produce individuals with rare anomalous colorations. Using a non-invasive camera trap technique, we recorded and described for the first time a case of a reddish tayra, *Eira barbara*, a coloration anomaly known as erythrism. In addition, we conducted a study based on the scientific literature on anomalous-colored individuals of the family Mustelidae, one of the most diverse groups of carnivorous mammals in the world. In this study, we present the results of this investigation and discuss its taxonomic, evolutionary, and ecological implications, as well as its applicability from a conservational perspective.

## 1. Introduction

Mammalian coloration results from the production and distribution of pigments in the skin, hair, and eyes. Coat color is associated with many biological and ecological functions, such as concealment, physiological regulation, and communication intra- and interspecifically [1,2]. The coloration is the result of the joint action of more than 150 genes, and chromatic disorders arise mainly from an excess or deficit of pigments (e.g., melanin [2,3]). In wild animals, morphological and biological coat coloration issues are disregarded. Different alleles of one gene may result in different phenotypes, and similar phenotypes may be caused by mutations in the alleles of different genes. For example, a white coat can result from the action of several genes, namely, KIT and EDNRB (both associated with leucism, EDNRB also being linked to piebaldism), TYR (the albino locus), and STX17 (progressive graying) [4,5]. Furthermore, chromatic disorders in wild mammals are difficult to detect in situ and anomalous individuals are often misclassified or poorly recorded in the formal scientific literature [6,7]. The scarcity of tissue samples from many wild animals with anomalous colorations hinders biochemical analyses, making morphological data an essential tool for investigating and identifying color variants in nature.

Mustelidae is the largest and most diverse family within the order Carnivora, comprising 65 species across 22 genera that are distributed throughout nearly all regions of the world [8,9]. Mustelids are typically characterized by elongated bodies, short limbs, and long tails. The smallest member, the Least Weasel (*Mustela nivalis* Linnaeus, 1766), weighs just 25 g, while the largest mustelid, the Sea Otter (*Enhydra lutris* [Linnaeus, 1758]) can reach 45 kg [10,11]. Among mammals, mustelids stand out for their wide variety of coloration patterns, ranging from dark tones, such as black and brown, to lighter tones, such as pale yellow and white, including facial markings, bands, or patches. Color mutations have been reported in several mustelids. In domestic species, such as the American Mink (*Neovison vison* [Schreber, 1777]), artificial selection and breeding aims to produce a range of colors in captivity, with color patterns varying from brown, to black and white, to spotted and bluish gray (“sapphire”). Furthermore, other species, such as the Ermine (*Mustela erminea* Linnaeus, 1758), undergo seasonal coat color changes in response to environmental conditions, shifting from brown in the summer to completely white in winter [11,12].

The Tayra, *Eira barbara* (Linnaeus 1758), is suggested as the mustelid species with the highest frequency of chromatic disorders reported in the formal scientific literature [13]. Tayras are scansorial, medium-sized Neotropical mammals, with small, rounded ears, stiff facial vibrissae, and a large, slender, muscular body (body length = 56–71 cm; body mass = 2.7–7 kg). They have a slightly humped back, limbs which are mostly elongated when compared to other mustelids (e.g., *Galictis* spp.), and a bushy tail, which is about two-thirds as long as their body (tail length = 36.5–46 cm) [13,14]. Tayras are widely distributed from Mexico to northern Argentina and occur mainly at lower altitudes in montane tropical forests [11,15]. They display a predominantly diurnal activity pattern, being an opportunistic omnivorous and feeding on invertebrates, small vertebrates, eggs, a variety of ripe and decaying fruits, and honey [15,16].

Tayras have a short coat, with soft underfur. The species’ coloration pattern is marked by shades of dark brown to black on the back, limbs, and tail, whereas the head and neck can be grizzled tan, greyish brown, pale yellow, or, more rarely, dark brown. The body’s ventral region is dark brown or black, with a bright or pale yellow-to-orange spot, often triangular, on the chest and throat. Juvenile tayras are commonly black and may have a white throat patch, a white head, or a dark mid-dorsal stripe extending to the tail. Some coat color variations in *E. barbara* are attributed to subspecies, with individuals exhibiting lighter to darker shades of brown on the body and black, white, or yellow on the head, neck, or tail [13]. Atypical colorations in tayras are commonly related to hypopigmentary disorders, mainly leucism (e.g., [17,18,19]), although other color anomalies are still poorly understood in this species.

In this study, we described the first case of erythrism in *Eira barbara*, a chromatic disorder that causes reddish to reddish-brown pigmentation in the tissues of vertebrates, including the skin, feathers, and hair. This anomaly is commonly associated with a recessive mutation in the Melanocortin 1 receptor (MC1R) (extension gene), leading to an overabundance of pheomelanin (red pigments) relative to eumelanin (dark pigments) across many parts of the animal’s body [3,19]. Erythrism is poorly known in wild mammals when compared to other color anomalies, with reported cases in some taxa, such as primates Cercopithecinae Gray, 1821 [20,21], squirrels (*Sciurus niger* Linnaeus, 1758) [22], rodents (*Oxymycterus rufus* [Fischer, 1814]) [23], artiodactyls (*Tragulus napu* [F. Cuvier, 1822]) [24], leopards (*Panthera pardus* [Linnaeus 1758]) [25,26], and anteaters (*Tamandua mexicana* [Saussure, 1860]) [3]. Records of erythrism in wild mustelids are rare and, to date, have been restricted to only four species. Additionally, we conducted a comprehensive and historical (1890–2024) survey of erythrism and other chromatic disorders in mustelids, by reviewing the formal scientific literature, aiming to correct misidentified cases, and discussing the global emergence of these chromatic anomalies and the ecological effects associated with anomalous individuals in different environments.

## 2. Materials and Methods

### 2.1. Record Area of the Erythristic Individual of Eira barbara

**Figure 1:**
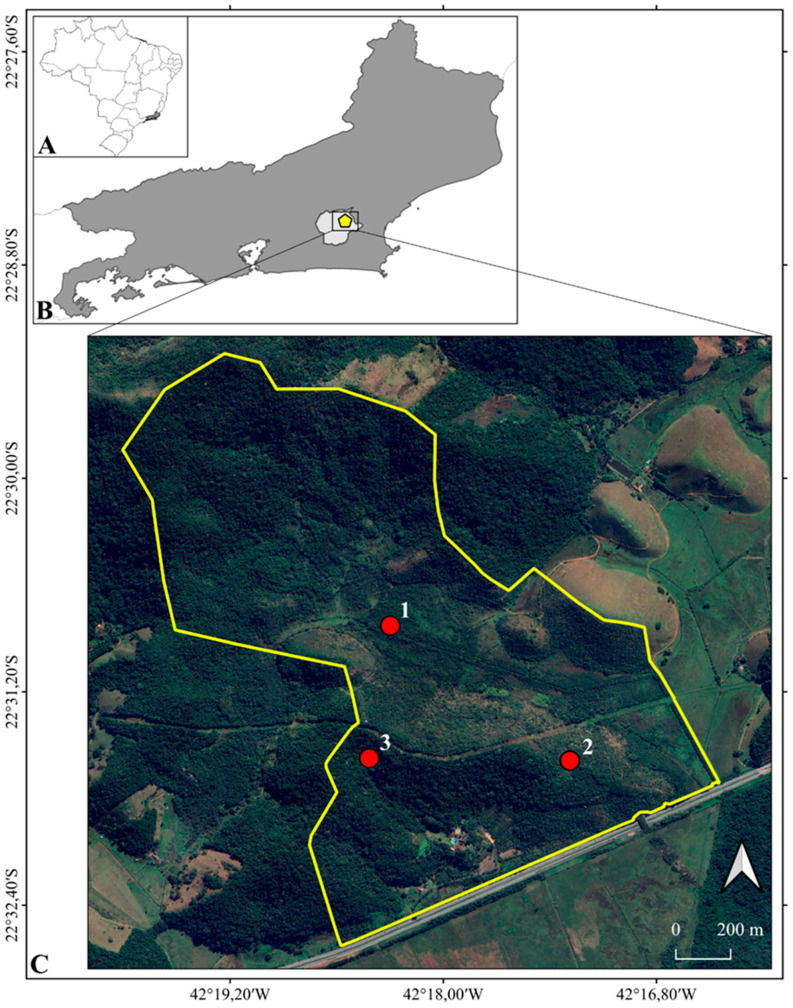
Location of erythrism records in *Eira barbara* in Rio de Janeiro state, Brazil (**A**), highlighting Silva Jardim municipality (**B**) and the Golden Lion Tamarin Ecological Park area (**C**). Source: Google Earth. The red circles 1, 2, and 3 represent the locations of the three erythrism records of *Eira barbara*.

The Parque Ecológico Mico-Leão-Dourado (PEMLD) (coordinates 22°30′37.44″ S and 42°18′27.03″ W) is a private natural heritage reserve that covers 237 hectares situated in the municipality of Silva Jardim, the state of Rio de Janeiro, southeastern Brazil. The study area lies in the coastal lowlands of the state and is part of the environmental protected area ‘Área de Proteção Ambiental da Bacia do Rio São João/Mico-Leão-Dourado’, a sustainable-use conservation unit (Figure 1). The region’s climate is humid tropical, with a dry winter season [27]. The terrain is characterized by plains with hills ranging from 30 to 200 m in altitude [28]. The forest areas are primarily Dense Lowland and Submontane Ombrophilous Rainforest (50–500 m) [29], with canopies that can reach heights of up to 30 m [30]. Historically, vegetation originally covered around 66% of the basin’s extension [31], but currently, the remaining forest fragments are surrounded by a heterogeneous matrix comprising regenerating and secondary forests, pastures, monocultures, and human settlements [32]. The region’s vegetation supports several threatened species, such as the Golden Lion Tamarin (*Leontopithecus rosalia* [Linnaeus, 1766]), endemic to the region, and the Southern Maned Sloth (*Bradypus crinitus* [Gray, 1850]) [33]. Furthermore, the study area is located within the Serra do Mar Ecological Corridor, a region of significant conservation importance, although frequently impacted by the presence of the federal highway BR-101, which separates this reserve from the Poço das Antas Biological Reserve (PA) [31,34]. Between 2018 and 2021, the Golden Lion Tamarin Association, a non-profit organization dedicated to the conservation of the Golden Lion Tamarin and its habitat, implemented reforestation efforts in areas formerly used as pasture in PEMLD, covering more than 80 hectares of the property previously known as Igarapé Farm [34].

### 2.2. Mammal Monitoring Survey by Camera Trap in Parque Ecológico Mico-Leão-Dourado

**Figure 2:**
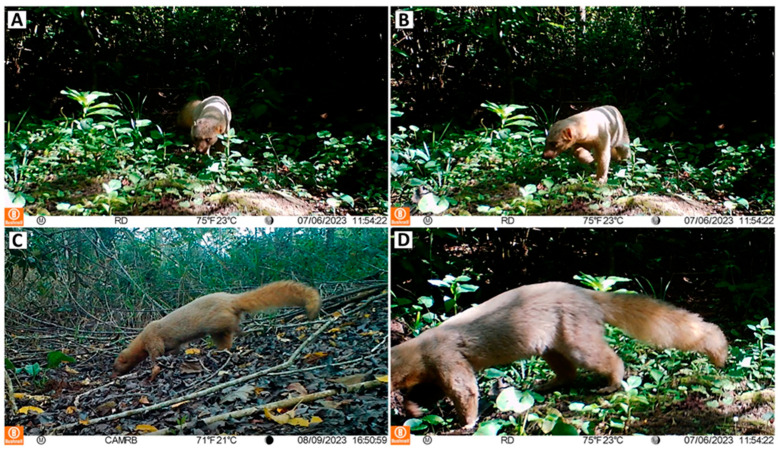
Records of the erythristic individuals of *Eira barbara* captured by camera traps. (**A**,**B**) show a mix of bright- and pale-red hairs on the head and ears, along with blackish hairs on the rostral portion of these individuals; (**C**,**D**) show a pattern of whitish-pink and grayish-pink hairs on the body and bright reddish hairs on the tail of these individuals. Each photo’s bottom bars display details about the sampling sites (RB = Restoration B; RD = Restoration D), temperature in degrees Celsius and Fahrenheit, moon phase, date and time of recording, respectively.

The mammal monitoring program carried out in the study area began in December 2022 with the activation of eight camera traps (Bushnell Core DS-4K No-Glow model 119987C; Bushnell; Overland Park, KS, USA), which were retrieved in June 2024. One single camera was used per sampling point, with a total of four cameras being placed in mature forested areas (>30 years) and four in reforested areas (5–6 years) (Appendix A - Table A1). The selection of camera location points was based on accessibility and the strategic placement of cameras aimed at maximizing species detection, along trails or open spaces near them. The cameras were set to operate 24 h a day, recording up to 30-s videos with a 1-s delay after motion detection and a 10-s interval between recordings. The cameras were typically positioned on tree trunks, at a height of 20 to 40 cm, and the angle of inclination was adjusted, when necessary, based on local conditions. Minimal vegetation disturbance was maintained, but in some cases, clearing was needed to prevent unnecessary camera triggers. No bait was used. The camera traps were inspected every 30 to 60 days for cleaning, checking battery levels, and downloading videos. Records of the same species were considered independent if they occurred more than one hour apart, regardless of the number of individuals in each recording (Figure 2).

Sampling effort was calculated based on the number of active camera-days, defined as the period between the first and last recording for each sampling month. Days when cameras malfunctioned or were removed were excluded from the calculation (Appendix A - Table A1). Capture Success (CS) was calculated as the ratio of independent records to sampling effort, multiplied by 100. This methodology was adapted from protocols used in other wildlife monitoring studies using camera traps [35].

### 2.3. Survey of Chromatic Disorders in Mustelidae in Formal Scientific Literature

In this study, we carried out an extensive review of chromatic disorders in mustelids, addressing the formal scientific literature by searching the following databases until September 2024: Web of Science, SciELO, Scopus, and Google Scholar. The investigation used a combination of keywords related to the taxonomic hierarchy (class, genus, and species names) and the terminology used to describe the most common chromatic disorders in mammals and other vertebrates, such as ‘albinism’, ‘leucism’, ‘melanism’, ‘piebaldism’, ‘xanthism’, ‘xanthochromism’, ‘erythrism’, ‘flavism’, ‘isabelism’, and ‘brown’. As seen in other studies on coloration in vertebrates (e.g., [3,36]), we considered that some names used to describe some chromatic disorders (e.g., flavism and isabelism) are often mistakenly applied to appoint anomalies that correspond to other chromatic disorders, such as erythrism and xanthochromism. Both disorders are commonly caused by a decrease in eumelanin (dark pigments) and an increase in pheomelanin (light pigments). Despite the historical misuse and lack of consensus regarding these nomenclatures among authors documenting mammalian coloration anomalies, we chose to include these terms in our search to account for their long-lasting presence in the scientific literature.

This literature search was conducted in English, Spanish, and Portuguese, initially comprising a total of 1349 scientific publications (articles or books) on coloration and chromatic anomalies in mammals. Publications that were mistakenly retrieved in our search (e.g., referring to other taxa or duplicate publications) were excluded from the total sample. Additionally, given that some mustelid species have significant commercial value (e.g., *Mustela putorius* Linnaeus, 1758) and that breeders often exploit color-related mutations, we also excluded articles related to chromatic disorders in captive animals. Following these exclusions, we focused solely on publications addressing chromatic disorders in wild mustelids. Seasonal color variations (e.g., changes occurring only in winter or summer) were not classified as chromatic disorders and were therefore not included in this survey.

The species of mustelids exhibiting anomalous coloration, along with their location records, vegetation profiles, and ecological traits, are presented in Appendix A - Table A1. Any possible misidentifications of chromatic disorders identified in the original publications were also reported in this table and discussed in the results section of this article. The “number of records” in Appendix A - Table A1 includes both sightings of individuals in the wild and specimens examined from scientific collections.

**Figure 3:**
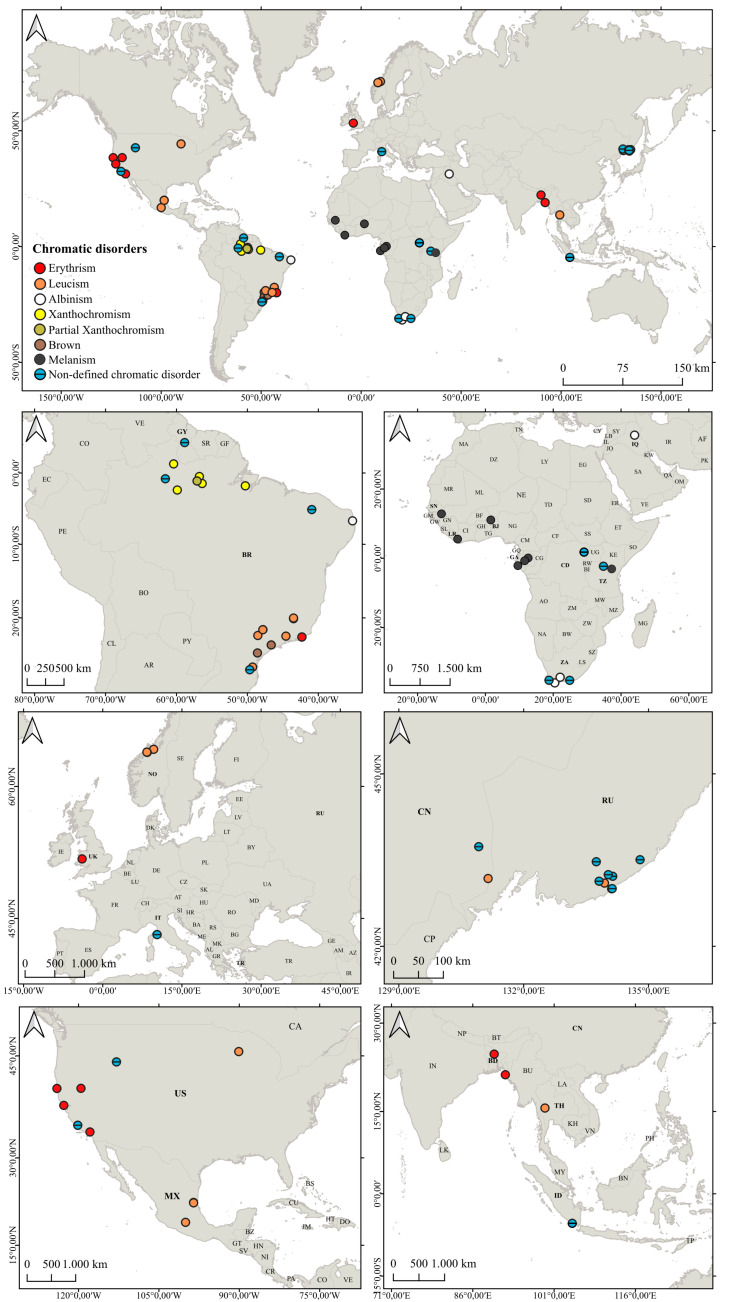
Map reviewing the distribution of chromatic disorders in Mustelidae worldwide.

The map displaying the global distribution of chromatic disorders in Mustelidae, based on our review, was produced using QGIS software version 3.28.10 (Figure 3).

## 3. Results and Discussion

### 3.1. Records of Erythrism in Eira barbara

The erythristic individual of *Eira barbara* reported here was identified during a fauna monitoring program (authorized under SISBIO license nº. 89707) conducted in reforested and mature forest areas within the Parque Ecológico Mico-Leão-Dourado (PEMLD), a particular protected area in the municipality of Silva Jardim, Rio de Janeiro, Brazil.

The mammal monitoring program carried out involved a total sampling effort of 2430 camera-days, resulting in 968 independent records of mammals from a total of 1127 videos (CS = 39.83). Among these, *Eira barbara* was recorded 18 times, corresponding to a CS of 0.74 for the species. Of these, 15 individuals displayed the typical coloration of the species, and three records were made of anomalously colored individuals.

These anomalous individuals showed striking reddish tones throughout their bodies (Figure 2). The head, ears, and neck of these individuals were covered by a mix of bright- and pale-red hair. In contrast, some blackish hairs were present from the lower nasomaxillary portion of the nostrils to the proximal portion of the upper lip, gradually reducing posteriorly, forming a subtriangular spot on the lateral surface of the rostrum. The laterosuperior portion of the rostrum, in turn, displayed the same reddish color observed in other parts of the head. The nostrils and eyes were black, as commonly observed in the species. The body had a distinct coloration from the head and neck. The coat from the withers to the rump presented a mixture of whitish-pink and greyish-pink hairs, with this pattern extending to the ventral region and limbs. The tail was covered in bright reddish hairs, slightly more evident and homogeneous than the coloration of the head and neck. The paws had a whitish-pink coloration, with slightly lighter tones when compared to the rest of the body. This reddish coloration pattern markedly contrasts with the typical color pattern of other individuals of this species.

Coat coloration in *Eira barbara* is often used as a marker of intraspecific variation (e.g., [13]). In the past, 16 subspecies of *E. barbara* were recognized based on body size and coat color [37]. Currently, only seven subspecies with unique coloration patterns are considered valid [11,38], namely, *Eira barbara barbara* (head [H] = gray to brown; body [B] = dull brown; throat patch [TP] = yellowish); *Eira barbara sinuensis* ([H] = not formally described—NFD); [B] = black; [TP] = may be present); *Eira barbara poliocephala* ([H] = NFD; [B] = dull brown; [TP] = yellowish); *Eira barbara peruana* ([H] = NFD; [B] = dark chocolate brown with legs darker than body; [TP] = NFD); *Eira barbara senex* ([H] = grayish white; [B] = dark brown; [TP] = absent); *Eira barbara inserta* ([H] = dark brown; [B] = black; [TP] = absent); and *Eira barbara madeirensis* ([H] = dark chocolate brown; [B] = chocolate brown darker than the head; [TP] = may be present). Some authors (e.g., [37]) argue that these subspecies may not be valid, since morphotypes with distinct body size and coat color lack geographic isolation, and these morphological differences might be better explained by climatic and sexual variation. However, regardless of the taxonomic status of these subspecies, the reddish individuals reported here do not match the coloration patterns of any of these supposed taxa. Instead, the coloration observed in the reddish individual of *E. barbara* here described is consistent with the distinct chromatic patterns seen in mammals with erythrism. For example, the pinkish-white coloration on the paws is a feature also identified in the skin of anteaters [3] and leopards [20] with erythrism. Although the nose, eyes, and some rare hairs are black, the overall coloration of the body reinforces the diagnosis of erythrism.

Two of our erythrism records occurred in restored areas (5 years old): one on July 6, 2023 (22°30′22.2″ S 42°18′17.7″ W) and the other on 9 August 2023 (22°30′05.6″ S 42°18′39.3″ W). The third record was obtained in a mature forest (>30 years old) on 4 April 2024 (22°30′22.56″ S 42°18′42.39″ W). Based on the morphological pattern and apparent age of the animal (likely an adult), we conservatively assumed that all these sightings represent the same individual with atypical coloration. However, we also cannot rule out the possibility that these records represent more than one individual exhibiting a similar color pattern. The distance between these locations varied between 500 and 850 m. Regardless, these sightings have always recorded a solitary animal, with no other *E. barbara* individuals nearby.

Our findings suggest a 16.6% frequency of tayras with erythrism in the study area, assuming an equal probability of detecting erythristic individuals and normally colored individuals in the same region.

### 3.2. Emergence of Erythrism in Eira barbara

The emergence of chromatic disorders in wild populations is still poorly understood, but they are frequently linked to inbreeding and environmental disturbances that occur in natural habitats [39,40]. In this study, we found that chromatic disorders in *E. barbara* were mostly documented in fragmented and human-modified areas (Appendix A - Table A1). Habitat loss and fragmentation reduce the home range of wildlife and promote population isolation, which, in turn, favors the decline in genetic diversity. The resulting genetic bottleneck can contribute to the emergence of anomalous colorations [41,42,43]. As such, human-mediated impacts on the habitat of the erythristic tayra may have been key factors promoting the emergence of anomalies such as the one reported here.

The erythristic tayra was mostly recorded moving through a fragmented landscape in the lowland Atlantic Forest. The greater incidence of light in the open canopy of restored areas where this animal was observed may have favored camouflage, possibly aiding its survival. Coat coloration plays an important role in mammalian camouflage, with darker fur usually being more common in tropical forest species since lighter coats could lead to increased visibility to predators and hunters or even reduce matting chances [1]. Some authors suggest that animals with chromatic disorders (e.g., albinism) may have a reduced life expectancy when compared to those with typical coloration [44]. However, the erythristic tayra appeared to be in good health, lacking obvious physical and/or developmental deficiencies, although its overall fitness compared to the rest of the *E. barbara* population remains uncertain. Nonetheless, it is worth emphasizing that other erythristic mammals, such as carnivores (e.g., [20,45]) and anteaters (e.g., [3]), appeared to be well adapted to their environments, all being adults with no observable and obvious health conditions. Furthermore, some visual impairments, such as blindness, previously observed in animals with other chromatic disorders (e.g., albinism and xanthochromism [46]) did not appear to occur in the erythristic *E. barbara* individual, and they have not been documented in other erythristic mammals described in the literature.

These records highlight the emergence of previously undocumented chromatic disorders in wild populations of *Eira barbara*. The first record of erythrism in this species highlights the importance of continuous monitoring of anomalies in wild animals, which is crucial for better understanding the broad implications of environmental stressors on wildlife. Biological and ecological data collected from these variants can be used as valuable indicators of population health and environmental disturbances, particularly in areas subject to frequent anthropogenic impacts.

### 3.3. Occurrence of Chromatic Disorders in Mustelids Worldwide

The survey of chromatic disorders presented in this study identified 34 studies with documentation of 119 mustelids with anomalous colorations among the 1349 formal scientific publications (articles and books) analyzed. We provide the results obtained from our survey below. Chromatic disorders are presented according to their original identification in the formal scientific literature, and subsequently, potential cases of misidentification of these anomalous individuals are discussed. Suggestions for reclassifying these cases under different chromatic disorders are provided alongside these results and in Appendix A - Table A1.

#### 3.3.1. Erythrism

Records of erythristic mustelids are scarce in the formal scientific literature and are often mistakenly classified as animals with other chromatic disorders, such as xanthochromism. In addition to our report of erythrism in *Eira barbara*, 10 erythristic individuals from four species of mustelids have been documented in the formal scientific literature (Appendix A - Table A1).

Two reddish-colored individuals of the European Polecat, *Mustela putorius*, were observed around Aberystwyth, Cardiganshire, UK, and compared with captive animals of the same species [47]. In addition, the author reports a population of erythristic animals from the same region. An erythristic individual of Long-Tailed Weasel, *Neogale frenata*, Lichtenstein 1831 (Syn: *Mustela frenata*) was observed in the Dickey Bird and Mammal Collection (no. 7574), at the University of California, Los Angeles, USA [48]. This specimen exhibited a reddish coloration with spots of varying shades on the back, rump and a spot above each ear. This animal was collected on 14 April 1918, in Covina, Los Angeles County, California. Another erythristic *N. frenata* individual was reported from Carlotta, Humboldt County, CA, USA. This specimen in question is markedly lighter in color than the other one mentioned above, with an ochre or faint reddish coloration that is nearly white.

Other mustelid species have also been reported to have erythrism. An erythristic individual of the American Badger, *Taxidea taxus* (Schreber, 1777)*,* was identified with a marked reddish coloration across its entire body [45]. This individual was found near the California–Nevada state boundary, T41N, T173, Sec. 10, Mount Diablo Meridian, Washoe County, Nevada, USA. The exact date of record is unclear, but it is assumed to be from the same year as the publication. Another possible erythristic specimen of *T. taxus* (no. 19744, Museum of Vertebrate Zoology, University of California, Berkeley, CA, USA) was collected in Marin County, CA, USA [49], about 483 km southwest from the site where the previous specimen was found [45]. Originally described as “pale reddish brown”, this specimen’s pinkish-brown pigmentation substituted the species’ typical black or brown coat. The body stripes were predominantly light brown, except for the usual white stripes on the head, common in badgers [45]. Contrary to the usual coloration in the species, where the limbs and face are typically blackish, this erythristic individual also displayed light-brown coloration in these areas. The claws retained their brown coloration, not differing from the coloration in other *T. taxus*. The ventral region was mostly yellowish, except for a white spot on the middle portion of the belly.

Additionally, four erythristic individuals of the Greater Hog Badger, *Arctonyx collaris* Cuvier, 1825, were reported between 2014 and 2021 in the regions of Jamalpur and Cox’s bazar, Southeast Bangladesh [50]. These animals were described as having a reddish or cinnamon-colored coat. Although the original article [50] did not include any photographs of the specimens, a photo of one of the animals was available through a link to a social media platform (Facebook) provided by the authors in their publication. The animal, photographed by Dhrubo Ahmed, exhibited an orange-reddish patch in the throat area, extending along the flanks to the dorsoposterior region of the proximal portion of the forelimbs, with a similar coloration in the tail. Its body hair was a very pale orange, almost yellow, and its limbs were dark brown. A dark-brown dorsal patch extended from the middle of the back to the top of the individual’s head. The individual’s skin had a light, slightly pinkish tone. From our observations, mustelids with erythrism can present a wide range of color tones, ranging from intense red, to reddish brown, to reddish orange, to pinkish orange, to pale red, and even to pinkish white. The skin is generally light-colored in erythristic individuals, with only some darker areas (e.g., the nostrils). Therefore, we recommend that both the skin and the coat should be examined to avoid misidentification of this chromatic disorder.

#### 3.3.2. Albinism

Albinism is a chromatic disorder historically reported in captive mustelids (e.g., [51,52]) but is rare in wild individuals.

An albino *Eira barbara* individual was recorded in the surroundings of Itatiaia National Park, Mantiqueira Mountain, Rio de Janeiro, Brazil [53]. This individual has a yellowish-white coat with cream tones covering most of its body and orange-yellow fur on its tail. The skin on its nostrils, lips, and claws are light pink. The animal’s eyes were closed in the photograph presented in that article, preventing a full color analysis. Based on its overall coloration, we consider that this animal may actually be leucistic rather than albino. However, due to the inability to assess its eye color, we have retained the original identification proposed by the authors. A young male albino Neotropical Otter, *Lontra longicaudis* (Olfers, 1818), was also reported from the Camaratuba River, Paraíba state, northeastern Brazil [54]. This individual had a very light yellowish-white coat, with light-pink skin and nails. Its eyes exhibited evident depigmentation of the iris, consistent with typical albino traits.

Only a few other records of albino mustelids have been reported in the formal scientific literature. A completely white individual of the Honey Badger, *Mellivora capensis* Storr, 1780, was recorded by camera traps in the De Hoop Nature Reserve, South Africa [55]. The animal was identified as albino based on its apparently pink ears and nose, in addition to its white coat, typical of this disorder. Another albino Honey Badger, with pink eyes and skin, was found on a farm in Prince Albert District, South Africa, and was later translocated to Anysberg Nature Reserve on 13 February 2020. Two additional informal records of white *M. capensis* were commented on in the same publication [55], but due to imprecise identification, these albinism records were not included in this review.

On 15 June 2022, a young albino Eurasian Otter, *Lutra lutra* (Linnaeus, 1758), was found in the Tigris River at Ishaqi District, south of Salah-Adain Province, Central Iraq [56]. This individual had mostly white fur, pink skin, a pink nose, pink feet, white claws and vibrissae, and red eyes, which are all characteristic traits of albinism.

An albino individual of the Hairy-Nosed Otter, *Lutra sumatrana*, was observed at Tanjung Belimbing near Way Haru, within the Bukit Barisan Selatan National Park’s environmental protection area in Sumatra, Indonesia, between 7 and 24 March 1997 [57]. However, the authors provided no visual evidence of this anomalous individual nor did they offer any detailed description of its phenotype. Additionally, the location data were limited and imprecise. As such, this record was not included in our survey.

#### 3.3.3. Partial Albinism

Albinism is characterized by the absence or inactivity of the tyrosinase enzyme, preventing the production of melanin throughout the life of affected animals. Consequently, the concept of “partial albinism” is often considered inadequate in the current scientific literature (e.g., [36]). Many anomalous conditions previously described as “partial albinism” were, in fact, other hypopigmentary disorders, such as leucism and piebaldism [3,6]. A so-called “partially albino” Eurasian Otter (*Lutra lutra*) was reported in the Lazovsky Nature Reserve, Primorsky Territory, Russia [58]. The animal was described as mostly white, with some black spots on the anterolateral portion of the belly, the side of the left hind limb, and the lower portion of the tail, also exhibiting depigmented eyes. However, after reviewing the photograph provided in the publication, we did not identify ocular depigmentation. Instead, the eye color seemed to be a typical shade of brown for the species rather than depigmented as initially suggested. We also question the classification of this animal as “partially albino” and propose that this record likely represents a leucistic *L. lutra* individual instead.

#### 3.3.4. Leucism

Leucism is one of the most frequently identified chromatic disorders in wild mammals, including mustelids. However, many of these records initially attributed to leucism may actually correspond to other chromatic disorders.

An individual of *Eira barbara* with anomalous coloration was recorded approximately 80 km from Manaus, Amazonas, Brazil, and identified as leucistic [59]. However, the animal has a strong orange coloration across most of its body, except for the black fur on its paws and facial mask. This pattern is not characteristic of hypopigmented mammals, such as leucistic ones. Instead, it aligns more closely with the coloration seen in animals with a predominance of yellow or orange pigments (e.g., pheomelanin), in their tissues, similar to that seen in xanthochromic individuals. In fact, the coloration of this animal [59] was very similar to that observed in anteaters with xanthochromism from several parts of northern South America [3], often including a black facial mask. Other similar cases of anomalous coloration were found in the scientific literature [17]. Thirteen *E. barbara* individuals from different regions of Brazil were identified as leucistic animals based on their yellowish coloration. However, among these, six specimens from Pará state had a golden-yellow coloration, mixed with brownish or black spots on their body or face, two of which had a well-marked facial mask (UFMG: 2981; MZUSP 5186). On the other hand, four other individuals from São Paulo state reported in the same publication [17] had yellowish-brown fur with dark-brown or black paws and claws. These brownish specimens resemble brown variants of other mammals (e.g., [3]) more than leucistic *E. barbara* individuals. The term “brown” is used in birds to describe individuals with an incomplete synthesis of melanin [36], a reduction in eumelanin and, consequently, brown body regions that should be expectedly black. The same term can be applied to mammals with similar anomalous brown coloration, such as anteaters [3]. Here, we use “brown” to identify the chromatic disorder in these *E. barbara* individuals.

An anomalously colored *E. barbara* individual was identified in Caracaraí, Roraima state, Brazil, whose coloration was previously attributed to leucism [60]. This animal had a very light-orange coloration but with black fur on the medio-distal third of its limbs and facial mask. Although described as “snow-white”, the proximal portion of the animal’s tail and the anterior portion of its hind limbs and head exhibited very discreet orange tones, appearing to be whitish due to intense sunlight reflecting off its fur. This coloration pattern is more characteristic of xanthochromism than the assigned leucism.

Other *Eira barbara* leucism records presented in our survey [[18,19,61]; see Appendix A - Table A1 for details] involve animals with pale-yellow coats, lighter and darker tones in different body parts, and some blackish undercoats. In addition, some cases also show slightly grayish fur on the distal third of the limbs and on the paws, mixed with the pale-yellow fur previously mentioned (e.g., [17], in the original article, Figure 1D; [61].

Leucism has also been reported in other mustelids. Three leucistic individuals of *Lontra longicaudis* were found in Temascaltepec, Mexico [40]. Although these specimens were taxidermized, two showed a homogeneous light-yellow coat, with only black nostrils and lips (possibly their eyes were likely dark when alive). Another specimen had a grayish-brown coat on most of its body but pale-yellow fur on the head and the inner (medial) regions of the limbs and a yellowish stripe extending from the medio-distal portion of the back to the distal portion of the tail’s dorsal surface. This animal, killed in 1981, may have acquired the yellowish coloration during taxidermy for integration into a collection. Since the specimen’s coat photo was not detailed enough for a refined analysis of its coloration, we maintained the original classification of leucism, though it could also be a leucistic or partially xanthochromic animal.

All other leucistic individuals reported in our study (Appendix A - Table A1) exhibited all-white coats and black eyes, nostrils, and, in some cases, claws. This pattern is the most commonly found in leucistic mammals. The only exception is an individual of the Yellow-Throated Marten, *Martes flavigula* (Boddaert, 1785), from the Northeast Tiger and Leopard National Park, Northeast China [62], which had pale-yellow fur and light-pink skin. This yellowish coloration was lighter and paler than the golden-yellow commonly seen in xanthochromic mammals and distinct from the orange-yellow typically found on the anteromedian portion of this species’ body. Thus, we agree with this identification [62] and classify it as leucism.

Additionally, two cases of leucism in *Meles meles* were reported from Central Norway [43], with eight other cases observed over a 10-year period in the same area. Since only details and images of the first two cases were available, we included them in our table and noted the others in this section due to the scarcity of information about them.

#### 3.3.5. Melanism

Melanism in wild mustelids is rarely reported in the formal scientific literature. All documented records correspond to the Honey Badger, *Mellivora capensis* (Appendix A - Table A1), with most reports originating from West and Central Africa [63,64,65,66]. Only one record of a melanistic individual was reported for East Africa, in the Ngorongoro Crater region, Tanzania [67].

All these melanistic individuals were described as entirely black animals, the typical trait of this chromatic disorder.

#### 3.3.6. Non-Defined Chromatic Disorder

Some mustelids with anomalous coloration are reported in the formal scientific literature without clear identification of the chromatic disorder that affects them, or with conflicting or mistaken classifications between authors. Thus, when precise identification was not possible, we classified these cases as “non-defined chromatic disorder” (Appendix A - Table A1).

An entirely yellow *Eira barbara* individual was reported during a mammal inventory in the Xixuau Nature Reserve, on the western side of the middle Rio Jauaperi, Roraima state, Brazil [68]. However, the author did not provide any details about the animal’s skin and fur color, making it difficult to determine whether the individual was leucistic or xanthochromic. Another *E. barbara* individual with a white-yellowish coloration was found in the State Biological Reserve of Sassafrás, Santa Catarina, Brazil [69]. This animal exhibited a light-yellow coloration across most of its body, with only a light-orange coloration on the upper portion of its neck and the mid-distal portion of the tail. A similar white-yellowish *E. barbara* was reported in the Serra das Almas Private Natural Heritage Reserve, Ceará, Brazil [70]. The coloration of this animal is very similar to the individual previously reported [69], with slightly more yellowish fur compared to the previously described specimen. The coloration pattern of these animals is consistent with that of leucistic *E. barbara*. However, due to insufficient photographic evidence of their skin and eye colors, the possibility of albinism cannot be ruled out.

Six sightings of yellowish *Eira barbara* individuals were also reported in Guyana [38]. These individuals exhibited a golden-yellow coloration and a black facial mask, with a chromatic pattern very similar to that observed in other specimens of *E. barbara* [17]. These individuals are likely xanthochromic, as suggested for other individuals with hypopigmentary conditions analyzed in the “Leucism” Section of this article.

Other mustelids with depigmentary/hypopigmentary conditions have been documented in the formal scientific literature. An individual of *Taxidea taxus* with anomalous coloration was found near the Pine Canyon Guard Station along California State Highway, in Santa Barbara County, along the Cuyama River, California, USA [71]. This animal was described as having a cream-colored coat from head to tail, with pale cinnamon tones on the legs and spots on the head and paws, being identified as a case of “partial albinism”. Some authors [45] have commented that this individual’s coloration resembles an anomalously colored specimen of *Neogale frenata* found in Carlotta, Humboldt County, CA, USA [48] (see the “Erythrism” Section in this article). Due to the lack of access to a color record of this individual, it was impossible to confirm the chromatic conditions (hypopigmentation and light spots) originally described for this specimen in the original article [71]. In this context, and considering other observations [45], this is likely a case of erythrism.

Another individual of *N. frenata* also presents an anomalous chromatic condition [48]. This animal is reported as a young male collected on August 6, 1890, in Birch Creek, Idaho, USA, and later deposited at the National Museum of Natural History, Smithsonian Institution (no. 23493), being identified as erythristic [48]. However, this animal is described as having a yellowish coloration rather than the reddish tones typical of erythrism, suggesting misidentification of this chromatic disorder. In fact, the description in the original publication [48] is indicative of a possible xanthochromic or leucistic condition. Considering the inconsistency in the information on this specimen and our inability to verify its coat coloration during this investigation, we chose to classify it as a non-defined hypopigmentary disorder (see Appendix A - Table A1).

A very white individual of the Small-Clawed Otter, *Aonyx cinereus* (Illiger, 1815), was found in Bukit Barisan Selatan National Park, Sumatra, Indonesia [72]. The animal’s tail appears to be the only dark part of the body, with brownish hairs on its mid-distal portion. However, due to sunlight exposure in camera trap records, it is possible that the tail appears dark due to shadows generated during its movement. Hence, we suggest this is either a leucistic or albino individual.

The occurrence of nine leucistic *Lutra lutra* individuals was reported from the Lazovsky Nature Reserve, Primorsky Territory, Russia [58]. These would add to the anomalously colored individual identified here as leucistic (see the “Leucism” Section above). However, insufficient information on their coat patterns prevents a clear distinction of their depigmentary/hypopigmentary condition.

Eight individuals of *Martes flavigula* with anomalous colorations were also reported [62]. Unlike the leucistic specimen described by the same author (see the “Leucism” Section above), these individuals exhibited various spot patterns, subdivided into types A–D. Type E corresponds to the leucistic animal previously described, being the remaining as follows: Type A = presence of white patches on the head, white forelimbs, and light brown mixed with white hair in the lumbar region; Type B = white head, with several gray spots on the forehead, and white forelimbs; Type C = gray hair on the snout and in part of the forehead, with black hind feet and white hair on the head, ears, tail, and thighs; Type D = white hair on all body parts where black hair is otherwise typical for this species. *Martes flavigula* has a typical yellow-orange “vest” from the lower portion of the head to the medio-distal portion of the body, limited anteriorly to the lumbar region, extending to the proximal portion of the forelimbs and throughout the belly. In these anomalous individuals [62], this vest has a bright-yellow to light-yellow coloration, distinct from the orange tone observed in the common coloration pattern of this species. The marked yellow coloration of these individuals and partial/total absence of dark coloration in the fur indicates a low production of melanin and suggests an overabundance of yellow pigments, i.e., partial xanthochromism. The spots’ asymmetrical pattern reinforces this hypothesis.

Whitish individuals of *Mellivora capensis* were registered across South and East Africa [65]. However, details on coat and skin coloration are lacking, preventing differentiation between leucism and albinism. It is interesting to note that a previously reported whitish *M. capensis* [73] was described as larger than typical for its species, possibly suggesting a mature animal that survived longer in the wild despite its higher susceptibility to predation due to its conspicuous white coloration.

Historically, some black specimens of *M. capensis* were originally considered as potential new species, currently included in the subspecies *Mellivora capensis cottoni* (Syn.: *Mellivora capensis sagulata*) [74,75,76]. However, this classification was questioned (e.g., [76]), and it was suggested that the color variations could be due to individual features, such as age. In fact, it is suggested that the white stripe typically found on the back of this species tends to fade with age, often resulting in older individuals appearing completely black [75,76]. We consider the chromatic condition of these all-black *M. capensis* individuals to be uncertain; they may be true melanistic individuals or simply older animals whose white fur has diminished with age, mimicking a coloration similar to that seen in anomalous forms. Therefore, we suggest that the identification of melanistic individuals of *M. capensis* should be approached with caution, taking into account ontogenetic variations before confirming potential chromatic anomalies in this species.

Finally, two anomalously colored European Pine Marten, *Martes martes* (Linnaeus 1758), were found on Elba Island, Central Italy [77]. These individuals displayed a light-yellowish pigmentation across their coats, appearing almost white, with light-pink skin (only nostrils and the pinna visible in the photos) and black eyes. The coloration pattern of these animals strongly aligns with that of leucistic animals. However, the photograph of one of these individuals was taken from a distance, preventing a detailed analysis of some body parts (e.g., belly and paws), whereas the other was captured under sunlight, making it difficult to accurately distinguish the shades of yellow in its fur. Therefore, we cannot rule out the possibility that these individuals have other pigmentary disorders, such as xanthochromism.

In addition to the cases reported above, two specimens of *Neogale frenata* were identified as “partially albino” [48]. One specimen (no. 223880, US National Museum, Billy’s Island) had a white nose and orbital perimeter, a white postorbital band, white tufts of hair on the antero-dorsal margin of the ears, and a white patch along the mid-dorsal line of the posterior region of the ears. The other specimen (no. 177679, US Nat. Mus., Billy’s Island) had a brown winter coat associated with white patches on the nose, right side of the neck, right hindleg, right foreleg, and tail tip. These specimens exhibited a coat pattern commonly observed in individuals with piebaldism, a chromatic disorder characterized by asymmetric, unpigmented (white) spots on the body, often linked to mutations in genes such as KIT, S Locus, and EDNRB, although the genetic basis is not uniform (e.g., [78,79,80]). However, considering the seasonal color changes in this species, we were unable to accurately determine the true color condition of these specimens based solely on the descriptive information provided in the original article [48]. Due to the limited data presented in the publication and the high likelihood that these were not cases of chromatic disorders, we opted not to include these records in Appendix A - Table A1.

#### 3.3.7. Prevalence of Chromatic Disorders in Mustelidae and Misidentification in Formal Scientific Literature

Our survey found that records of mustelids with anomalous coloration accounted for 8.82% (N = 119) of the data collected in the formal scientific literature, distributed in 35 of the 1349 analyzed publications. Chromatic disorders were reported across the Americas, Europe, Africa, and Asia (Figure 3) and identified in 14 mustelid species, with the following distribution: 21.84% (N = 26) melanism, 17.64% (N = 21) leucism, 10.92% (N = 13) erythrism, 7.56% (N = 9) xanthochromism, 3.36% (N = 4) albinism, 2.52% (N = 3) brown, and 36.13% (N = 43) non-defined chromatic disorders (Appendix A - Table A1).

**Figure 4:**
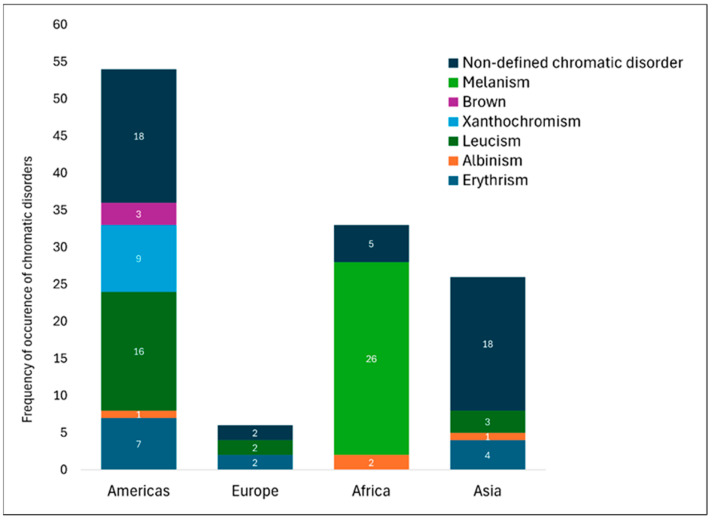
Frequency of occurrence of chromatic disorders in Mustelidae worldwide.

It is important to emphasize that while records of mustelids with melanism are the most numerous, they are restricted to Africa, with no formal reports in the formal scientific literature from other regions of the world. The highest prevalence of chromatic disorders was observed in the Americas; representing 45.37% (N = 54) of the records attributed to anomalous mustelids (Figure 4). Among these American records, 29.62% (N = 16) were leucistic; 16.66% (N = 9) were xanthochromic/partially xanthochromic; 12.96% (N = 7) were erythristic; 5.55% (N = 3) were anomalously brown; 1.85% (N = 1) were albino; and 33.33% (N = 18) were of non-defined chromatic disorders. In addition, most of these anomalously colored records originated in South America (81.48%, N = 44). However, we emphasize that these numbers possibly do not reflect the real prevalence of chromatic disorders in nature.

Studies on anomalous colorations in mammals are still incipient when compared to other groups, such as birds (e.g., [36,81]), and are even scarcer for taxa like Mustelidae. Additionally, numerous records of chromatic disorders are informally disclosed, either on social media platforms (e.g., Facebook) or scientific-record-sharing websites (e.g., iNaturalist). The absence of these records from the formal scientific literature means that much information remains inaccessible to the broader scientific community. In addition, many formally published records lack thorough review, often containing misidentifications of chromatic disorders or outdated terminology to describe these anomalies.

Finally, some recent studies have proposed new nomenclature for identifying chromatic disorders in vertebrates (e.g., [6,36]). Although we agree with some of the terms suggested for anomalously colored mammals (such as the use of “brown” in this paper), we recommend caution in their application. Grouping certain chromatic disorders under a single designation can be misleading, since these conditions may have distinct genetic origins. For example, melanism in mammals is commonly associated with mutations in genes linked to eumelanin biosynthesis, such as the melanocortin 1 receptor (Mc1r) and the K Locus [82], resulting in an increased production and distribution of this pigment. The “brown” condition [36], in turn, results from incomplete eumelanin synthesis, which produces different shades of brown in affected animals. Therefore, grouping both brown and black phenotypes under the term “melanism”, as proposed in some studies [6], seems inaccurate. Moreover, some chromatic disorders in mammals are strongly linked to the distribution of subpopulations of some species, such as leopards with erythrism in South Africa [20] and anteaters with leucism and xanthochromism in the Amazon [4], being potential bioindicators of the emergence and stabilization of anomalous conditions in the environment. In this context, grouping several anomalous phenotypes under a single term like “hypomelanism” [6] can generate inaccurate interpretations and retrieval of imprecise biological, ecological, and geographic information. This issue becomes even more critical when analyzing specimens from zoological collections, where storage conditions can alter coat coloration over time. For instance, leucistic individuals acquire a markedly yellowish hue that is easily confused with that of xanthochromic animals. Thus, using single terms to report these disorders can mask relevant information about anomalous phenotypes that are region-specific, making the identification of environmental imbalances difficult (e.g., habitat fragmentation and inbreeding). We acknowledge that the nomenclature of chromatic disorders in mammals has been historically inconsistent, with various names often attributed to the same anomalies and no consensus reached among authors. We suggest that phenotypic investigations should be complemented with genetic data obtained in the current scientific literature (e.g., [83]) to improve the accuracy of chromatic disorders’ identification. This approach also avoids the continued use of outdated or inaccurate terms, such as “partial albinism”, for future cases involving the identification of color anomalies in vertebrates, particularly mammals.

#### 3.3.8. Ecological Effects of Chromatic Disorders in Wild Species

The emergence of chromatic disorders may have different ecological effects on wild mustelid populations worldwide. In fact, color anomalies are often associated with different genetic, metabolic, and/or environmental factors, including excess or deficiency in melanin production, poor diet, exposure to pollution, habitat fragmentation, restricted gene flow, increased inbreeding, and reduction in genetic diversity [84,85]. Hypopigmentary disorders, such as leucism and albinism, often negatively impact affected individuals, increasing their susceptibility to predation, reducing their attractiveness for mating, and leading to frequent intraspecific exclusion, causing them to be quickly removed from wild populations [1,86]. Albinism is particularly rare, with some authors [87] suggesting that its frequency is less than 2% in abundant taxonomic groups, such as rodents. In contrast, albino populations of wild animals have historically been reported in some parts of the world. In Southeast Asia, for example, there are records of isolated groups of certain species, including the Slow Loris, *Nycticebus coucang* (Boddaert, 1785); the Bornean Orangutan, *Pongo pygmaeus* Linnaeus 1760; and the Sumatran Mountain Maxomys, *Maxomys hylomyoides* Robinson and Kloss 1916 [3].

In mustelids, albinism is less frequently recorded compared to other chromatic disorders (e.g., melanism and leucism), suggesting that it may be rare in wild populations, as mentioned above. Considering that albino animals commonly have visual anomalies due to ocular depigmentation, this may be a limiting factor for their survival by making them more vulnerable to predators during daily activities (e.g., foraging). This may also explain the greater number of records of leucistic and xanthochromic mustelids in our survey. Although these animals have light coloration, making them more exposed to predators, such as albinos, the absence of visual impairments may provide increased fitness by improving their predator detection. However, we cannot rule out the possibility that other congenital disorders might affect the survival chances of light-colored individuals. For example, in other species, such as the Big-Eared Opossum, *Didelphis aurita* Wied-Neuwied, 1826, a case of partial xanthochromism has been associated with other anomalies, namely, cortical blindness, hyperactivity, and self-injurious behavior (SIB) [45]. Thus, it is possible that these hypopigmented animals are more susceptible to fitness decline compared to hyperpigmented individuals, such as those with melanism. Furthermore, the occurrence of hypopigmented wild mammals may be more frequent in areas with high human activity [85]. In this context, it is suggested that environmental stress and inbreeding derived from habitat fragmentation and decreased population connectivity are factors that favor the emergence of color mutations. For instance, an albino Giant Anteater *Myrmecophaga tridactyla* Linnaeus, 1758 [88], was reported in a region of Brazil where high levels of inbreeding and low genetic diversity were recorded [89]. We conjecture that the scarcity of research on chromatic disorders in Mustelidae may conceal similar situations in other species.

In contrast, melanism seems to promote adaptive advantages for certain species, occurring in up to 20% of some populations [1]. In Felidae, for example, melanic polymorphism is common and present in 11 species worldwide. This color mutation is linked to different deletions in the MC1R gene in some felids, such as the Jaguar, *Panthera onca* Linnaeus, 1758, and the Jaguarundi, *Herpailurus yaguarondi* (Saint-Hilaire, 1803) [90], whereas in the Domestic Cat, *Felis silvestris catus* (Linnaeus, 1758), the mutation is caused by a two-base pair deletion in exon two of the agouti gene [91]. Some authors [92,93] suggest that melanic felids are more active during the day and gain a cryptic advantage over their prey during bright nights and lunar light, benefiting from superior camouflage when inhabiting dense forests. This gives them a fitness advantage when hunting during brighter conditions compared to non-melanic individuals of the same species (Temporal Segregation Hypothesis). Furthermore, melanic polymorphism in felids is also associated with climate variables (temperature and humidity; Gloger’s rule), with these variants presenting increased fitness in tropical forests when compared to open grasslands or savannas and being favored by natural selection due to more efficient thermoregulation and camouflage in shaded areas [93]. Some felids, such as jaguars, have markings (e.g., white patches in their ears) that play a role in intraspecific communication but are absent in melanistic individuals. It has been suggested that while non-melanistic felids benefit from better short-range communication, they also face greater conspicuousness. This trade-off is thought to be balanced by the adaptive advantage of better camouflage in melanistic felids, favoring the evolutionary persistence of melanic polymorphism in wild populations [93,94]. In other mammals, such as anteaters, a high incidence of brown and black individuals have also been observed in tropical forests, such as the Amazon [3]. In mustelids, these patterns remain less understood, but it is likely that melanic polymorphism also provides advantages, such as more efficient camouflage and thermoregulation, similar to those observed in Felidae. Although melanic mustelids have only been documented in Africa so far, it is likely that many cases are unreported or are informally recorded, leading to a loss of valuable data on chromatic disorders.

Erythrism is a less frequently reported chromatic disorder in mammals and, consequently, has been less studied from an ecological perspective. However, erythristic individuals recorded in the formal scientific literature are typically adult animals, apparently healthy and showing no apparent physical or mental deficiencies. Although rarer than melanism, erythristic mammals may have a better survival advantage than hypopigmented individuals. The recent sighting of erythristic leopards in East and South Africa [20,26] and erythristic anteaters in Panama [3] reinforces the fact that these color morphs can survive in both open and closed areas. While they may not have the same camouflage efficiency as darker-colored animals, they are likely to be more successful than light-colored anomalous individuals. However, information about this and other chromatic disorders in mustelids is still scarce and requires further studies to be better clarified.

We suggest that chromatic disorders in mustelids should also be addressed from a conservation perspective. Although most species within the Mustelidae family are currently classified as “Least Concern” on the IUCN Red List, recent data indicate that 31 species were experiencing global population declines, with some, such as the European Mink, *Mustela lutreola* (Linnaeus, 1761), now classified as “Critically Endangered” [95]. That said, the identification of anomalous colorations can serve as a valuable bioindicator for detecting ecological imbalances that may be potentially impacting wild mustelid populations. For instance, a reduction in the presence of color morphs, such as melanistic forms, may signal advanced deforestation in tropical forests, as these morphs are often favored by natural environments.

## 4. Conclusions

The identification of color disorders in mustelids, as in many other mammals, remains a challenging task. The lack of standardized nomenclature for these anomalous conditions often results in the use of erroneous or obsolete terms when reporting anomalously colored animals in the formal scientific literature. In this study, we present the first recorded case of erythrism in *Eira barbara* and provide a comparative review of the terminology and formal records of chromatic anomalies in mustelids worldwide. Many records of color disorders that are not formally documented in the scientific literature are dispersed across informal media platforms, such as social networks (e.g., Facebook) and scientific-record-sharing sites (e.g., iNaturalist). We reinforce the importance of publishing these records and providing accurate morphological, ecological, and geographic data on mustelids with anomalous coloration as well as other mammalian taxa.

## Data Availability

The original contributions presented in this study are included in this article; further inquiries can be directed to the corresponding authors.

## Appendix A

**Table A1:** Chromatic disorders for Mustelidae in formal scientific literature (1890–2024).

Original Chromatic Disorder Identification	Reviewed Chromatic Disorder Identification	Species	Record Area	Number of Records	General Profile of Record Areas	Date	Reference
**Erythrism**	**Erythrism**	*Eira barbara*	Parque Ecológico Mico-Leão-Dourado (PEMLD), municipality of Silva Jardim, Rio de Janeiro, Brazil (22°30′22.2″ S 42°18′17.7″ W; 22°30′05.6″ S 42°18′39.3″ W and 22°30′22.56″ S 42°18′42.39″ O)	3 *	Mature (>30 years) and reforested (<5 years) areas of lowland Atlantic Forest	6 July 2023; 9 August 2023; 4 April 2024	This article
		*Taxidea taxus*	Near the California–Nevada state boundary, T41N, T173, Sec. 10, Mount Diablo Meridian, elevation 4700 feet (1430 m), Washoe County, Nevada, USA	1	Oak woodland mixed with chaparral area	No date (possibly 2006)	[45]
			Marin County, CA, USA (region about 300 miles [ca. 483 km] southwest from the site where the specimen reported by Laacke et al. 2006 was found)	1	Mix of conifers, hardwood, herbs, with regenerating or shrubby trees	No date	[49]
		*Neogale frenata oregonensis*	Carlotta, Humboldt County, CA, USA	1	Coast Redwood forest area	20 December 1930	[48]
		*Neogale frenata latirostra*	Covina, Los Angeles County, CA, USA	1	Coastal sage scrub area	14 April 1918	
		*Mustela putorius putorius*	District around Aberystwyth, Cardiganshire, United Kingdom	2 ^†^ (with description of a wild population in the region)	Coastal salt-tolerant plants; deciduous and mixed woodlands; upland heathlands and moorlands; and riparian vegetation areas	1902 and 1903 (the first two records)	[47]
		*Arctonyx collaris*	Jamalpur and Cox’s bazar, Southeast Bangladesh	4 ^‡^	Tropical partial evergreen forests (Cox’s bazar) and deciduous forest (Jamalpur)	January 2014 (Jamalpur); January 2020, August 2021, and July 2021 (Cox’s bazar)	[50]
**Albinism**	**Leucism**	*Eira barbara*	Surroundings of Itatiaia National Park, Mantiqueira Mountains, Rio de Janeiro, Brazil (22°23′24.71″ S, 44°33′52.16″ W)	1	Mature and reforested Montane Atlantic Forest area, in different regeneration phases	July 2015	[53]
	**Albinism**	*Lutra lutra*	Watercourse of the Tigris River at Ishaqi District, 20 km to the northwest of Balad Township, south of Salah Adain Province, Central Iraq (34°03′56.3″ N 44°01′48.2″ E)	1	Mesopotamian Shrub Desert Ecoregion, riparian forest area	15 June 2022	[56]
	**Albinism**	*Mellivora capensis*	De Hoop Nature Reserve, South Africa (34°27′06.34″ S, 20°29′05.11″ E)	1	Scrubland vegetation (fynbos) areas	21 November 2020	[55]
	**Albinism**	*Mellivora capensis*	Farm in the Prince Albert District, South Africa (33°04′08.43″ S, 22°02′59.91″ E)	1	Scrubland vegetation (fynbos) areas	13 February 2020	[55]
	**Albinism**	*Lontra longicaudis*	Camaratuba River, near the Biological Reserve Guaribas, municipality of Mamanguape, Paraíba state, northeastern Brazil (6°43′ S, 35°10′ W)	1	Seasonal semi-deciduous forest and savanna area	September, 2013	[54]
**Partial albinism**	**Leucism**	*Lutra lutra*	Lazovsky Nature Reserve, Primorsky Territory, Russia (43°01′17″ N, 134°07′54″ E, elevation 18 m)	1	Oak riparian area	29 October 2018	[58]
**Leucism**	**Leucism**	*Eira barbara*	Rio Vermelho Municipal Environmental Protection Area, Santa Catarina, Brazil	1	Savanna relics mixed with tropical forest regions impacted and fragmented by large crops	June 2015	[19]
	**Xanthochromism**	*Eira barbara*	Caracaraí, Roraima, Brazil (1°17′24.6″ N, 60°23′56.5″ W)	2 ^†^	Human settlement (fish-bone pattern of deforestation in Amazon forest area)	13 February 2008; 16 February 2008	[60]
	**Leucism**	*Eira barbara*	R.P.P.N Amadeu Botelho Ecological Reserve (22°18′24″ S, 48°31′26″ O), São Paulo, Brazil	3 ^†^	Fragmented region with only 1.5% of native seasonal semi-deciduous, deciduous, and riparian forests; the fragments are only 30 ha and are distant from one another (Atlantic Forest)	11 October 2018; 5, February 2019; 3 March 2019	[18]
	**Leucism**	*Eira barbara*	Jataí Ecological Station, São Paulo, Brazil (21°32′27,5″ S, 47°48′09,3″ W and 21°33′06,5″ 6,5″ S, 47°49′12,0″ W)	2 ^†^	Agricultural and fragmented landscape, sugarcane field, and paved road surrounding native vegetation of Atlantic Forest	May 2013; August 2013	[61]
	**Leucism**	*Eira barbara*	Private natural heritage reserve (R.P.P.N.) Santuário do Caraça, Catas Altas, Minas Gerais, Brazil (20°0′51″ S, 43°29′28″ W)	2 ^†^	Impacted semi-deciduous forest area (Atlantic Forest)	11 January 2009; 22 February 2009	[17]
	**Leucism**	*Eira barbara*	Private natural heritage reserve (R.P.P.N.) Santuário do Caraça, Minas Gerais, Brazil	3 ^‡^	Impacted semi-deciduous forest area (Atlantic Forest) with intense mining activities	12 June 2009; 17 July 2012; March 2016	
	**Xanthochromism**	*Eira barbara*	Rio Trombetas, Oriximiná, Pará, Brazil	1	Museum specimen (MPEG 9210) collected in an impacted area of the Amazon Rainforest with intense mining activities	15 July 1978	
	**Partial xanthochromism**	*Eira barbara*	Cachoeira Porteira, Oriximiná, Pará, Brazil (1°06′57.20″, S 57°05′24.50″ W)	2 ^†^	Museum specimens (MPEG 10013 and MPEG 10014) in an impacted area of the Amazon Rainforest with intense mining activities	4 April 1977; 26 July 1978	
	**Xanthochromism**	*Eira barbara*	Porto Trombetas, Oriximiná, Pará, Brazil (1°28′20.0″, S 56°22′35.5″ W and 1°28′20.0″, S 56°22′35.5″ W)	2 ^†^	Museum specimen (UFMG 2981) collected in an impacted area of the Amazon Rainforest with intense mining activities	No date; November 2007	
	**Xanthochromism**	*Eira barbara*	Boiuçú, Pará, Brazil (1°48′0.000″ S, 50°16′59.880″ W)	1	Museum specimen (MZUSP 5186) collected in an impacted area of the Amazon Rainforest with intense mining activities	26 April 1965	
	**Brown**	*Eira barbara*	Jardim Público de São Paulo (current Parque da Luz), São Paulo, Brazil	2 ^†^	Museum specimens (MZUSP 2061 and MZUSP 2062) collected in Jardim da Luz, São Paulo, Brazil; possibly brought from another Brazilian region and introduced into the park	November 1905; December 1905	
	**Brown**	*Eira barbara*	Iporanga, São Paulo, Brazil	1	No data	28 January 1944	
	**Xanthochromism**	*Eira barbara*	80 km from the city of Manaus, Amazonas, Brazil (2°23′23.65″ S, 59°52′45.96″ W)	1	Secondary forest with 20 years (Amazon Rainforest)	25 June 2014	[59]
	**Leucism**	*Lontra longicaudis annectens*	Río Temascaltepec (19°04′33″ N, 100°02′42 W, elevation [el.] 1747 m); Guayalejo River at the ejido Tantoyuquita (22°32′41″ N, -98°33′10″ W, el. 10 m); near an irrigation channel close to the town of Nueva Apolonia (22°28′240″ N, -98°32′49″ W, elevation 20 m); all in Temascaltepec municipality, state of México	3 ^‡^	Cropland and riparian forest area	Winter 1981; December 2008; December 2011	[40]
	**Leucism**	*Pekania pennanti*	Private land near Brantwood, Wisconsin, U.S.A. (45°33′0 45.82″ N; 90°06′54.75″ W)	1	Mixed deciduous forest area	9 September 2017	[7]
	**Leucism**	*Meles meles*	30 May and 19 August 2017 in Orkdal (63°19′52.6″ N 9°38′31.7″ E); 26 March to 18 August 2018 close to Halsa (63°04′04.1″ N 8°25′05.2″ E; 63°05′18.5″ N 8°21′24.6″ E), Central Norway	2 ^†^	Mountainous forest area	30 May 2017; 19 August 2020	[43]
	**Leucism**	*Martes flavigula*	Northeast Tiger and Leopard National Park, Northeast China	1	Coniferous and mixed broadleaf forest area	13 and 18 January 2018	[62]
	**Leucism**	*Lutrogale perspicillata*	Watercourse at Huai Kha Khaeng Wildlife Sanctuary, Thailand	1	Riparian forest area	Inaccurate date (video posted in 2020)	[56] (Link: https://www.youtube.com/watch?v=ItqhSfCBtmQ (Accessed on 12 September 2024)
**Melanism**	**Melanism**	*Mellivora capensis*	Niokolo-Koba National Park, Senegal	13 ^‡^	Mix of grassland, bush, and woodland savannas; floodplains; and riparian vegetation areas	March to June 2021	[65]
	**Melanism**	*Mellivora capensis*	Ivindo National Park and Loango National Park, Gabon	2 *	Lowland equatorial rainforest areas	2002 to 2006	[63]
	**Melanism**	*Mellivora capensis*	Putu Iron Ore Mining exploration concession on the eastern slope of Mont Jideh in the Putu Mountains, southeastern Liberia	3 ^‡^	Mature and dense rainforest areas	Between December 2010 and April 2011	[64]
	**Melanism**	*Mellivora capensis*	Gabon	1	Mix of rainforest, semi-deciduous forests, and grass-dominated savanna areas	2003	[65]
	**Melanism**	*Mellivora capensis*	Pendjari National Park, Benin	6 ^‡^	Mix of grassland, shrub, wooded savanna, open and extensive gallery forests, as well as riparian and semi-deciduous forests	Between 20 February 2021 and 11 April 2021	[66]
	**Melanism**	*Mellivora capensis*	Ngorongoro Crater, Tanzania	1	Mix of savanna grassland, forest, and woodland areas	No data	[67]
**Non-defined chromatic disorder**	**Possible xanthochromism**	*Eira barbara*	Guyana	8 ^‡^	No data	No date	[38]
	**Possible xanthochromism**	*Eira barbara*	Serra das Almas Private Natural Heritage Reserve, Ceará, Brazil	1	Mixed of dense seasonal scrub, seasonal thorn forest, and seasonal montane deciduous forest	2013 to 2015	[70]
	**Possible leucism or xanthochromism**	*Eira barbara*	Xixuau Nature Reserve, Roraima, Brazil (S 0°48.023′, W 6l°33.476′)	1	Amazon Rainforest area	January 2001 to April 2001	[68]
	**Possible leucism**	*Eira barbara*	Sassafrás State Biological Reserve, Santa Catarina, Brazil (26° 42″ S, 49° 40″ W)	6 ^#^	Transition zone between tropical rainforest and mixed rainforest areas	September 2004 to November 2006	[69]
	**Possible leucism**	*Martes martes*	Elba Island, Italy (42°47′12″ N, 10°16′28″ E)	2 ^†^	Mediterranean maquis, with the presence of large oak forest areas	10 March 2020; 23 May 2020	[77]
	**Possible leucism**	*Lutra lutra*	Lazovsky Nature Reserve, Primorsky Territory, Russia (43°29′14″ N, 134°44′38″ E, elevation [el.] 393 m; 43°08′47″ N, 133°48′39″ E, el. 160 m; 43°00′49″ N, 134°06′51″ E, el. 22 m; 43°01′00″ N, 134°07′35″ E, el. 22 m; 43°31′20″ N, 134°47′47″ E, el. 468 m; 43°13′59″ N, 134°08′19″ E, el. 243 m; 43°15′40″ N, 134°01′54″ E, 370 m)	9 ^‡^	Oak riparian mix or broadleaf/pine riparian mix	Between 1980 and 2018	[58]
	**Possible leucism or albinism**	*Mellivora capensis*	Hankey, Cape Province, South Africa	1	Predominance of all vegetation types of the Fynbos Biome	No date	[73]
	**Possible leucism or albinism**	*Mellivora capensis*	Young honey badger brought from the wild to Tygerberg Zoo, Western Cape, South Africa, and then to the Cheetah Conservation & Research Centre, South Africa, in 2001; died in 2014 of old age at 16 years old	1	Natural area of origin of the individual not specified	2001 to 2014	[65]
	**Possible leucism or albinism**	*Mellivora capensis*	Serengeti National Park, Tanzania	1	Savanna areas	2009	[65]
	**Possible melanism**	*Mellivora capensis*	Eastern fringe of the Ituri Forest to a point fifteen miles west of Mawampiand thence southeast to Boni, Ghana, northeastern Congo, at elevations of between 2100 and 2950 feet above sea level	2 ^†^	Climax dense tropical rainforest area	No data	[65,75];
	**Possible leucism or albinism**	*Aonyx cinereus*	Bukit Barisan Selatan National Park, Sumatra, Indonesia	1	Mix of montane forest, lowland tropical forest, coastal forest, and mangrove forest areas	20 April 2015	[72]
	**Possible erythrism**	*Taxidea taxus*	Two miles northeast of Pine Canyon Guard Station on California State Highway 166, in Santa Barbara County, along the Cuyama River, California, USA	1	Mix of pine conifers, oaks, and chaparral; flat and dry valley	November, 1959	[71]
	**Possible partial xanthochromism**	*Martes flavigula*	Northeast Tiger and Leopard National Park, Northeast China	8 ^‡^	Coniferous and mixed broadleaf forest area	11 January 2015; 26 October 2016; 21 November 2016; 28 December 2016; 18 September 2012, 14 October 2012; 15 February 2020; 28 August 2020; 26 September 2020	[62]
	**Possible xanthochromism**	*Neogale frenata nevadensis*	Birch Creek, Idaho, USA	1	Mix of riparian vegetation, coniferous forests, and shrub areas	6 August 1890	[48]

* One individual with several sightings. ^†^ Two individuals sighted. ^‡^ Multiple individuals sighted. ^#^ Multiple sightings with an imprecise number of individual records.
